# An audit of some processing effects in aggregated occurrence records

**DOI:** 10.3897/zookeys.751.24791

**Published:** 2018-04-20

**Authors:** Robert Mesibov

**Affiliations:** 1 West Ulverstone, Tasmania, Australia 7315

**Keywords:** Atlas of Living Australia, GBIF, occurrence records, data errors

## Abstract

A total of ca 800,000 occurrence records from the Australian Museum (AM), Museums Victoria (MV) and the New Zealand Arthropod Collection (NZAC) were audited for changes in selected Darwin Core fields after processing by the Atlas of Living Australia (ALA; for AM and MV records) and the Global Biodiversity Information Facility (GBIF; for AM, MV and NZAC records). Formal taxon names in the genus- and species-groups were changed in 13–21% of AM and MV records, depending on dataset and aggregator. There was little agreement between the two aggregators on processed names, with names changed in two to three times as many records by one aggregator alone compared to records with names changed by both aggregators. The type status of specimen records did not change with name changes, resulting in confusion as to the name with which a type was associated. Data losses of up to 100% were found after processing in some fields, apparently due to programming errors. The taxonomic usefulness of occurrence records could be improved if aggregators included both original and the processed taxonomic data items for each record. It is recommended that end-users check original and processed records for data loss and name replacements after processing by aggregators.

## Introduction

Neither the Atlas of Living Australia (ALA) nor the Global Biodiversity Information Facility (GBIF) simply republishes the occurrence records it receives from data providers. Each aggregator processes incoming data in an effort to improve data quality. The processing works by adding, deleting or modifying data items, or by adding “assertions” (ALA) or “flags” (GBIF) to records that contain items identified as incorrect, incomplete, suspect or otherwise invalid.

Processing of this kind can be beneficial when data errors are corrected or flagged. Processing is not helpful when valid data items are lost and when added or modified data items are incorrect. To investigate some of the effects of processing I audited ca 345,000 occurrence records from the Australian Museum (AM) and ca 355,000 occurrence records from Museums Victoria (MV), in each case as the records appear in both ALA and GBIF. I was mainly interested in the changes made by the aggregators to taxon names, but I also checked for data losses in selected non-taxonomic fields, and for the latter purpose I examined ca 100,000 occurrence records in GBIF from the New Zealand Arthropod Collection (NZAC).

As reported below, some processing operations significantly downgraded rather than upgraded data quality, and changes in taxon names varied substantially between aggregators.

New processing routines are occasionally introduced by ALA and GBIF, and old ones improved over time (e.g., see the “Issues” section of the ALA “biocache-store” GitHub site, https://github.com/AtlasOfLivingAustralia/biocache-store). For this reason the results presented here should be seen as “date-stamped” early 2018, when I downloaded the sample data.

## Methods

### Data sources

From the ALA website I downloaded the “Australian Museum Malacology Collection” and “Museums Victoria provider for OZCAM” datasets. I chose Darwin Core (Wieczorek et al. 2012) downloads in TSV format with all fields in the record-class terms, occurrence, organism, event, location, identification, and taxon categories. From the ALA-MV dataset I selected all records with the *collectionCode* “Entomology”. Each ALA table contains both original and processed data.

From the GBIF website I downloaded Darwin Core archives containing both original (*verbatim.txt*) and processed (*occurrence.txt*) record tables for “Australian Museum provider for OZCAM” and “Museums Victoria provider for OZCAM”. From the AM dataset I selected original and processed records with the *collectionCode* “Malacology” and from the MV dataset the original and processed *collectionCode* “Entomology” records.

I also downloaded from GBIF ca 100,000 original and processed records from the New Zealand Arthropod Collection (NZAC). The NZAC dataset had internal data problems that prevented me from auditing its taxonomic content effectively, but some GBIF processing effects on non-taxonomic NZAC data are noted in Results.

Download or data citations as recommended by ALA and GBIF are as follows:

AM Malacology from ALA

Atlas of Living Australia occurrence download at https://biocache.ala.org.au/occurrences/search?&q=collection_uid%3Aco114 accessed on Wed Feb 14 18:44:13 AEDT 2018

AM from GBIF

Australian Museum (2017). Australian Museum provider for OZCAM. Occurrence Dataset https://doi.org/10.15468/e7susi accessed via GBIF.org on 2018-02-14

MV from ALA

Atlas of Living Australia occurrence download at https://biocache.ala.org.au/occurrences/search?&q=data_resource_uid%3Adr342 accessed on Wed Jan 31 06:42:40 AEDT 2018

MV from GBIF

Museums Victoria (2017). Museums Victoria provider for OZCAM. Occurrence Dataset https://doi.org/10.15468/lp1ctu accessed via GBIF.org on 2018-01-30

NZAC from GBIF

Wilton A (2018). New Zealand Arthropod Collection (NZAC). Version 1.67. Landcare Research. Occurrence Dataset https://doi.org/10.15468/lrgzz9 accessed via GBIF.org on 2018-01-08

After finding disagreements between ALA downloads and the ALA website (see Results and Discussion), I downloaded two additional record sets for checking, rather than auditing:

AM from ALA (standard download)

Atlas of Living Australia occurrence download at https://biocache.ala.org.au/occurrences/search?&q=collection_uid%3Aco114 accessed on Mon Feb 19 10:40:22 AEDT 2018

MV from ALA (standard download)

Atlas of Living Australia occurrence download at https://biocache.ala.org.au/occurrences/search?&q=collection_uid%3Aco39 accessed on Mon Feb 19 10:39:30 AEDT 2018

### Data auditing and preparation

I audited the records tables on the command line with BASH and GNU text-processing tools and GNU AWK 4 (Robbins 2018). The eight original working tables (AM and MV data from ALA; AM, MV and NZAC data from GBIF) have been archived in Zenodo (https://doi.org/10.5281/zenodo.1217733; version 2 uploaded 2018-04-13).

For convenience in cross-checking the results of ALA and GBIF processing, I reduced the AM and MV datasets to records with catalogNumber in common between ALA and GBIF, i.e. 345,944 AM records and 355,824 MV records.

The GBIF NZAC dataset had 1186 pairs of duplicate records, in each case with one record with *modified* date “2016-11-11” and the other with “2017-05-09” (see Results for a likely explanation). I deleted the earlier record versions, reducing the NZAC dataset to 102,092 records.

### Issues with field structuring

Although the ALA and GBIF downloads both contain original and processed data, direct comparisons are not straightforward because of the way the aggregators have structured and filled their data fields. ALA, for example, has duplicated or pseudo-duplicated five of its download fields. Simple duplicates are *basisOfRecord* (two fields) and *recordedBy_raw* (three fields). *dcterms:bibliographicCitation* (two fields) is pseudo-duplicated, with different entries in the two replicates (noted in another ALA download; the fields are blank in the two downloads audited here). There are two *class* fields, and ALA explains in the *headings.csv* file included in the download archive that one *class* field contains “Class matched / The class the ALA has matched this record to in the NSL [National Species Lists] http://rs.tdwg.org/dwc/terms/class”, while the other is only explained as “http://rs.tdwg.org/dwc/terms/class”. A check of MV data indicates that the second *class* field contains original data items, and it is surprising that ALA does not label this field *class_raw* (as it has done with *kingdom_raw*, *phylum_raw*, *order_raw*, *family_raw* and *genus_raw*). The fifth duplicated field is more problematic. ALA generates two *specificEpithet* fields and one *specificEpithet_raw* field, with the following explanations in *headings.csv*:

*specificEpithet* = “Species matched / Original scientific name supplied with the record http://rs.tdwg.org/dwc/terms/scientificName”

*specificEpithet* = “http://rs.tdwg.org/dwc/terms/specificEpithet”

*specificEpithet_raw* = “http://rs.tdwg.org/dwc/terms/specificEpithet”

Contradicting the explanations, the first *specificEpithet* field is not a duplicate of *scientificName_raw* (also provided in the download), the second *specificEpithet* holds the originally supplied species name and is therefore actually *specificEpithet_raw*, and *specificEpithet_raw* is blank.

The ALA download also includes the confusingly named:

*verbatimDepth* = “http://rs.tdwg.org/dwc/terms/verbatimDepth”

*verbatimDepth_raw* = “http://rs.tdwg.org/dwc/terms/verbatimDepth”

*verbatimElevation* = “http://rs.tdwg.org/dwc/terms/verbatimElevation”

*verbatimElevation_raw* = “http://rs.tdwg.org/dwc/terms/verbatimElevation”

The two “raw” fields are empty in both the AM and MV datasets, but in another dataset I examined (National Herbarium of Victoria records, NHV; https://collections.ala.org.au/public/show/co55, accessed 2018-03-14) it is clear that the processing from *verbatimElevation_raw* to *verbatimElevation* is intended to convert elevations in units other than metres to elevations in metres. The “raw” entry “985ft”, for example, was processed as “300.228”. Where *verbatimElevation_raw* is already in metres in the NHV dataset, *verbatimElevation* either repeated the entry with added 0.1 m precision (e.g. “1616” becomes “1616.0”) or deleted the entry if it was not simply parseable (e.g. *verbatimElevation_raw* = “1627.000 m”, *verbatimElevation* blank). ALA processing also failed with range entries, e.g. “15000–17000 ft” was not converted to metres.

GBIF has not duplicated any fields or confused the field naming in its download, but *verbatim.txt* and *occurrence.txt* differ significantly in their field structure. The *associatedMedia*, *geodeticDatum*, *verbatimCoordinates*, *verbatimLatitude*, *verbatimLongitude* and *scientificNameAuthorship* fields are dropped without replacement during processing, for unknown reasons. The *country* field is dropped but its items are processed (with additions, corrections or exclusions) into *countryCode* in *occurrence.txt*. Minimum and maximum depth and elevation are recalculated by GBIF during processing. In *occurrence.txt*, *minimumDepthInMeters* and *maximumDepthInMeters* are replaced by *depth* and *depthAccuracy*, where “depth” is either the single depth value supplied, or the mean of the supplied minimum and maximum, and “depthAccuracy” is the average deviation from the mean. *minimumElevationInMeters* and *maximumElevationInMeters* are similarly replaced by *elevation* and *elevationAccuracy*.

GBIF adds *genericName* and *species* fields to its processed tables. The terms are defined by GBIF online (http://gbif.github.io/dwc-api/apidocs/org/gbif/dwc/terms/GbifTerm.html; accessed 2018-02-15) but neither term is part of the Darwin Core standard (see http://rs.tdwg.org/dwc/terms/). The first field is “The genus part of the scientific name”, yet in many MV records *genericName* contains a non-genus name. The *species* field contains “The canonical name without authorship of the accepted [processed] species” and seems to be the same as the *species* field in the recommended GBIF download. I ignored the *genericName* and *species* fields in the audit.

I also found that there are ALA fields populated with data items with the corresponding GBIF fields completely blank. These are not losses due to processing, since the fields are also blank in the *verbatim.txt* file. The field contents were evidently not supplied to GBIF, either by ALA, which acts as Australia’s GBIF node, or by the data provider. For example, AM *catalog Number* C.153619.002 appears on the ALA website (https://biocache.ala.org.au/occurrences/4b52f4f9-01e0-411c-adc5-213e40a40b0f; accessed 2018-03-15) and in the download with the *habitat* entry “On pink Aplysilla” (original and processed), but there is no *habitat* entry on the corresponding GBIF webpage (https://www.gbif.org/occurrence/1100892212; accessed 2018-03-15), and the *habitat* field in the GBIF-AM *verbatim.txt* file is blank. In the AM dataset the blanked fields are *acceptedNameUsage, associatedMedia, associatedOccurrences, class, dataGeneralizations, day, geodeticDatum, habitat, identificationRemarks, informationWithheld, month, nameAccordingTo, occurrenceStatus, otherCatalogNumbers, samplingProtocol, taxonConceptID, verbatimCoordinateSystem, verbatimEventDate, verbatimLatitude, verbatimLongitude, verbatimTaxonRank, waterBody* and *year*, and in the MV dataset *associatedMedia, class, dataGeneralizations, georeferencedBy, georeferenceProtocol, georeferenceSources, informationWithheld, nomenclaturalCode, samplingProtocol, taxonConceptID* and *waterBody*.

In all the fields I audited for processing changes, I found that the original data items (“raw” items in ALA, *verbatim.txt* items in GBIF) were identical in ALA and GBIF, i.e. there was no “cascading effect” of processing changes from ALA (as GBIF node) to GBIF.

### Taxon names

In examining name changes after processing I ignored changes in taxonomic authorship. Whether attached to names or entered in the *scientificNameAuthorship* field, authorships are often incomplete or incorrect in original records. As noted above, GBIF drops the *scientificNameAuthorship* field from processed records, instead adding authorship to some, but not all names in the *scientificName* field.

I also ignored processing changes in the higher classification of taxa, such as changes in family assignments for genera. Although changes in classification can make records harder to discover in a search of aggregated data, those changes reflect differences in the classification schemes used by data providers and aggregators, and might be regarded as matters of opinion by end-users of aggregated data. However, I used higher-taxon changes as guides when looking for incorrect replacements (see Results).

The search for processing effects on names was further limited to records with genus- or species-group *scientificName* in the original, and I excluded records in which the original *scientificName* was informal, e.g. “Idiosepius _n.sp._2”. Totals examined were 340,998 records in the AM dataset and 331,480 in the MV dataset.

Both ALA and GBIF attempt to match taxon names with names in reference classifications. GBIF uses a “backbone taxonomy” (https://www.gbif.org/dataset/d7dddbf4-2cf0-4f39-9b2a-bb099caae36c; accessed 18 January 2018) and ALA refers to Australian National Species Lists (https://www.ala.org.au/uncategorised/data-processing/; accessed 18 January 2018). Processing of *scientificName* could result in no change to the name supplied, or in one or more of the following outcomes, listed below with examples from ALA-processed records.

**deleted.** Name has no replacement; processing deletes it.

*Jaffaia jaffaensis* (Blochmann, 1910) (AM *catalog Number* C.100786)

**fail-match.** Name replaced with *incertae sedis*, with a name from an unrelated branch of the classification, or with an incorrect name, such as a homonym.

The trichopteran *Lasiocephala basalis* (Kolenati, 1848) (MV TRI43315) was matched to the plant taxon Drosera sect. Lasiocephala

**up-match.** Name generalised to one at a level in the taxonomic hierarchy above the supplied or appropriate one.

*Oliva parkinsoni* Prior, 1975 replaced with *Oliva* (AM C.100860)

**down-match.** Name particularised to one at a level in the taxonomic hierarchy below the supplied or appropriate one.

Arrenurus (Arrenurus) replaced with *Arrenurus madaraszi* (MV H14890107)

**swap-match.** Name replaced with another at the same rank. For ALA records, this category includes species-level names differing only in subgenus.

*Polyphrades brevirostris* Lea (MV COL100011) replaced with *Essolithna rhombus*

**subgenus.** Subgenus added to or deleted from species or subspecies name; no other major changes (ALA records only).

Vexillum (Costellaria) antonelli (Dohrn, 1861) (AM C.407864) replaced with *Vexillum antonellii*

**amended.** Only minor change to name spelling or format.

*Hasora discolor mastusia* Fruhstorfer, 1911 (MV LEP11) replaced with *Hasora discolora mastusia*

For each record in which the processed *scientificName* differed from the original *scientificName*, I tabulated *catalog Number*, original name, processed name, change type (one of the categories listed above), change detail and original *typeStatus*. An example from ALA:

T4607 | Culex (Lutzia) douglasi Dobrotworsky | Culex (Neoculex) douglasi | swap-match | species for species | Holotype

Obviously, a processed *scientificName* entry may represent more than one kind of change. For example, an up-matched taxon may also be a swap-match at the higher taxon level, as with *Anaxo cylindricus obscurus* Blackburn (MV T13669) up-matched to the synonym *Lepturidea cylindrica* by ALA. In the change tables, the ranking order for non-deleted names is fail-match > (up-match = down-match) > swap-match > subgenus > amended. Because GBIF does not usually include subgenera in processed names, the GBIF change tables do not include “subgenus”-type entries, and only a few changes involving original subgenera could be included in other categories. It is also likely that at least some of the up-, down- and swap-matched records are actually fail-matched (see Results).

The four name change tables for AM-ALA, AM-GBIF, MV-ALA and MV-GBIF are included in the Zenodo archive with the records downloads.

## Results

### Name changes: *ALA*

Including all change types, ALA changed formal names in the genus- and species-groups in 72,963 records in the AM dataset (21.4%) and 46,835 (14.1%) in the MV dataset (Table 1). Ignoring the less significant “subgenus” and “amended” change types, the totals are 62,824 (AM, 18.4%) and 38,374 records (MV, 11.6%.).

**Table 1.** Tallies of records with changes by ALA in genus- and species-group names in the AM and MV datasets. Totals of records with formal, genus- and species-group names: AM = 340998, MV = 331480.

AM:			MV:		
deleted	genus	116	deleted	genus	37
deleted	species	726	deleted	species	98
fail-match	genus for plant	2	fail-match	species for plant	1
fail-match	species for plant	22	down-match	genus to species	2
down-match	genus to subgenus	21	down-match	genus to subgenus	727
down-match	species to subspecies	2041	down-match	subgenus to species	2
up-match	genus to class	134	down-match	species to subspecies	1093
up-match	genus to order	1	up-match	genus to class	3
up-match	genus to family	1317	up-match	genus to order	83
up-match	subgenus to family	6	up-match	genus to family	4157
up-match	subgenus to genus	65	up-match	genus to subfamily	79
up-match	species to class	257	up-match	subgenus to genus	93
up-match	species to superfamily	2	up-match	species to class	2
up-match	species to family	8015	up-match	species to order	173
up-match	species to genus	21641	up-match	species to family	1575
up-match	species to subgenus	40	up-match	species to subfamily	159
up-match	subspecies to class	25	up-match	species to tribe	310
up-match	subspecies to family	244	up-match	species to genus	8973
up-match	subspecies to genus	397	up-match	species to subgenus	72
up-match	subspecies to species	1154	up-match	subspecies to family	68
swap-match	genus for genus	14	up-match	subspecies to genus	374
swap-match	species for species	26462	up-match	subspecies to subgenus	22
swap-match	subspecies for subspecies	122	up-match	subspecies to species	6107
subgenus	added to species	1980	swap-match	genus for genus	752
subgenus	deleted from species	338	swap-match	subgenus for subgenus	8
amended	species for species	7338	swap-match	species for species	11438
amended	subgenus for subgenus	212	swap-match	subspecies for subspecies	1966
amended	subspecies for subspecies	1	subgenus	added to species	3959
			subgenus	deleted from species	2083
			subgenus	added to subspecies	119
			subgenus	deleted from subspecies	134
			amended	genus for genus	3
			amended	species for species	1807
			amended	subspecies for subspecies	356
Total		72693	Total		46835

Names deleted in processing are missing from the standard ALA download but still appear on the ALA website. An example is the record for the onychophoran *Planipapillus bulgensis* Reid (MV K3033) at https://biocache.ala.org.au/occurrences/e96c0cd8-79ce-43ae-82b4-90f9a2d7d6ac (accessed 28 February 2018). The webpage displays the name and classification for this museum specimen lot, but the “original vs processed values” dialog box shows that the supplied *scientificName* has been filtered out, and this webpage is not found with a search in ALA for “Planipapillus bulgensis”. (GBIF did not delete or change any of the names deleted by ALA.)

The two records down-matched from genus to species (MV HET19158, HET19159) are for specimen lots of the moth *Praxis edwardsii*. MV supplied the *specificEpithet* “edwardsii” to ALA but omitted the epithet from *scientificName*. Some other down-matches are a little surprising as they occur not through replacement by a synonym, but within the same parent taxon. For example, the ant species *Pheidole bos* has three valid subspecies in Australia (https://biodiversity.org.au/afd/taxa/Pheidole/names; accessed 28 February 2018). MV records for *Pheidole bos baucis* (HYM46113) and *P. bos eubos* (HYM46138) have *taxonRank* originally specified as “subspecies” and are processed without change as subspecies. Five records for *P. bos* with *taxonRank* specified as “species” and with no subspecific name supplied are down-matched to *P. bos bos* (HYM46132-HYM46136) and re-ranked as “subspecies”. The down-matching noted in the Methods section, of Arrenurus (Arrenurus) to *Arrenurus madaraszi* (MV H14890107), is likewise hard to understand, as MV did not specify a species, the subgenus Arrenurus contains numerous species and *A. madaraszi* is placed in the subgenus Micruacarus. (GBIF did not down-match any of the names down-matched by ALA.)

### Name changes: *GBIF*

Including all change types, GBIF changed formal names in the genus- and species-groups in 50,080 records in the AM dataset (14.7%) and 44,519 (13.4%) in the MV dataset (Table 2). Ignoring the less significant “amended” change type, the totals are 47,453 (AM, 13.9%) and 37,124 records (MV, 11.2%.).

**Table 2.** Tallies of records with changes by GBIF in genus- and species-group names in the AM and MV datasets. Totals of records with formal, genus- and species-group names: AM = 340998, MV = 331480.

AM:			MV:		
fail-match	class to genus	1	swap-match	genus for genus	3021
fail-match	species for species	13	up-match	genus to family	101
swap-match	species for species	218	up-match	species to order	8
up-match	genus to family	30	up-match	species to family	169
up-match	species to phylum	2	up-match	species to genus	14957
up-match	species to family	261	up-match	subspecies to genus	368
up-match	species to genus	46900	up-match	subspecies to species	18500
up-match	species to subgenus	5	amended	genus for genus	3
up-match	subspecies to genus	1	amended	species for species	5875
up-match	subspecies to species	22	amended	subspecies for subspecies	1517
amended	genus for genus	53	Total		44519
amended	species for species	2574			
Total		50080			

GBIF deleted no names in processing. One fail-matched record is for AM *catalog Number* C.479173, identified as “Aplacophora” by AM and replaced by GBIF with the bivalve genus *Aulacophora* Jeffreys, 1882. The other fail-matches are for the marine snail names *Nuculana pala* (Hedley, 1907) (12 records) and Nuculana (Ledella) pala (Hedley, 1907) (one record), which were incorrectly matched with *Nuculana pella* (Linnaeus, 1758) (*pala*: http://www.marinespecies.org/aphia.php?p=taxdetails&id=506315, accessed 2018-03-03; *pella*: http://www.marinespecies.org/aphia.php?p=taxdetails&id=140578, accessed 2018-03-03). (ALA did not change “Aplacophora” in processing, and swap-matched the *pala* names to *Ledella pala*.)

### Name changes: *ALA* vs *GBIF*

Despite the roughly comparable numbers of name changes, ALA and GBIF processed the same set of names very differently. Table 3 tallies these differences as numbers of records. The overlap (names changed by both ALA and GBIF) is remarkably low. Further, among records with names changed by both ALA and GBIF there was substantial lack of agreement on the type of change (Table 4). However, for most of the records with both ALA and GBIF up-matching the original name, the two processed names were the same, with exceptions generally limited to genus differences (species up-matched to genus) or species differences (subspecies up-matched to species).

**Table 3.** Tallies of records in which either ALA or GBIF changed formal genus- and species-group names.

AM (340998 records):		
ALA only	ALA and GBIF	GBIF only
56123	16570	33510
**MV (331480 records)**:		
ALA only	ALA and GBIF	GBIF only
36467	10368	34151

**Table 4.** Tallies of name change types among records in which both ALA and GBIF changed formal genus- and species-group names.

ALA change type	GBIF change type	No. of records
AM dataset:		
deleted	up-match	34
fail-match	up-match	1
down-match	up-match	32
swap-match	swap-match	4
swap-match	fail-match	13
swap-match	up-match	5054
swap-match	amended	275
up-match	swap-match	7
up-match	up-match	8525
up-match	amended	573
subgenus	up-match	90
subgenus	amended	219
amended	up-match	1275
amended	amended	468
	Total	16570
MV dataset:		
down-match	up-match	12
down-match	amended	13
swap-match	swap-match	8
swap-match	up-match	700
swap-match	amended	330
up-match	swap-match	90
up-match	up-match	6795
up-match	amended	1076
subgenus	up-match	468
subgenus	amended	38
amended	up-match	80
amended	amended	758
	Total	10368

In the AM dataset, the four records swap-matched by both ALA and GBIF are for one species:

*Gyraulus coranus* (Iredale, 1943) (AM)

> Gyraulus (Gyraulus) essingtonensis (ALA)

> *Gyraulus corinna* (Gray, 1850) (GBIF)

The eight MV records swap-matched by both ALA and GBIF are for two species:

Lipotriches (Hoplonomia) (MV)

> Nomia (Hoplonomia) (ALA)

> *Hoplonomia* Ashmead, 1904 (GBIF)

Leioproctus (Nodocolletes) (MV)

> Leioproctus (Lamprocolletes) (ALA)

> *Nodocolletes* Rayment, 1931 (GBIF)

The 32 AM records with the same species-group name down-matched and up-matched are for:

*Erronea chrysostoma* Schilder, 1927

> *Erronea ovum chrysostoma* (ALA)

> *Erronea* Troschel, 1863 (GBIF)

The 12 MV records with the same name both down- and up-matched are for two species:

*Palaminus australiae* Fauvel, 1878

> *Palaminus australiae australiae* (ALA) [ALA here ignores a second subspecies, *P. a. hebridensis* Cameron, 1934]

> *Palaminus* Erichson, 1839 (GBIF)

*Dabra termitophila* Lea, 1906

> *Dabra termitophila termitophila* (ALA) [ALA here ignores a second subspecies, *D. t. victoriensis* Lea, 1910]

> *Dabra* Olliff, 1886 (GBIF)

### Name changes: type status

A consequence of name changes in processing is that a type specimen can lose its association with the name it represents. The AM and MV change tables include numerous records of primary types (Table 5). Among the large number of swap-matches, especially after ALA processing, there are types listed by the aggregator which are not, in fact, types of the replacement name. An example is MV T4295, the holotype of *Amaloptila triorbis* Turner, 1903, which in ALA has the processed synonym name *Elesma subglauca* Walker, 1865 with Type status = “holotype” (https://biocache.ala.org.au/occurrences/163727ac-8ba3-4dbb-a5c0-bf79d9474f04; accessed 2018-03-03). The holotype of *E. subglauca* is actually in the Natural History Museum (London) (http://www.nhm.ac.uk/our-science/data/butmoth/search/GenusDetails.dsml?NUMBER=9539.0; accessed 2018-03-03). (GBIF did not change “*Amaloptila triorbis* Turner, 1903”.)

**Table 5.** Tallies of name change types for primary type specimen lots (holotypes, lectotypes, neotypes, syntypes) among records with formal genus- and species-group names in the AM and MV datasets.

AM-ALA		MV-ALA	
deleted	12	deleted	27
down-match	20	down-match	28
swap-match	397	swap-match	1637
up-match	753	up-match	560
subgenus	16	subgenus	320
amended	57	amended	46
Total	1255	Total	2618
**AM-GBIF**		**MV-GBIF**	
fail-match	1	swap-match	3
swap-match	1	up-match	223
up-match	1326	amended	100
amended	48	Total	326
Total	1376		

When checking for fail-matches (see below), I noted an issue with AM types on the GBIF website. The AM specimen lot C.26622 for *Nuculana pala* (Hedley, 1907) is a holotype, as can be seen in the “Diagnostics” section of the relevant GBIF webpage (https://www.gbif.org/occurrence/1100962172, accessed 2018-03-03), but although the specimen lot has the processed *typeStatus* = “holotype” in *occurrence.txt*, “Type status” is blank on the webpage and the processed value has the remark “Excluded”.

### Name changes: unrecognised fail-matches

Without checking thousands of name changes individually, it was impossible to determine how many up-, down- and swap-matches resulted in a taxon name being replaced with one from another branch of biological classification, or with a non-synonym (see the GBIF *pala/pella* example, above). The 39 fail-matched records tallied in the change tables are the most obvious failures I found. I suspect there are many more, but using higher classifications in name-changed records as a guide was made impractical by unfilled higher-taxon entries (AM) and disagreements on higher taxa between data provider and aggregator (MV).

### Data losses

Aggregator processing sometimes results in loss of a data item: an original record contains a data item in a particular field, but after processing that field is blank for the record concerned. I found a surprisingly high number of data losses in the audited datasets. Below I give examples of loss (see also the comment above on deleted taxon names in ALA, and on verbatim depth and elevation data in Methods: *Issues with field structuring*). For more details, see *data_notes.txt* in the Zenodo archive for this project.

*identifiedBy*: AM-ALA and MV-ALA, 100% loss in processing. The original *identifiedBy_raw* data item appears on the ALA webpage as “Identified by” for the record but is missing from the standard (recommended) download).

*locality*: MV-ALA, 100% loss in processing. The original *locality_raw* data item appears on the ALA webpage as “Locality” for the record but is missing from the standard (recommended) download). (*locality_raw* is blank in the AM dataset.)

Losses of date information were common and evidently due to processing rules written to deal with various date formats. In the *modified* field in the NZAC dataset, for example, GBIF successfully parsed 4765 entries in YYYY-MM-DDTHH:MM:SS+12:00 format, but deleted 97,327 entries in YYYY-MM-DDTHH:MM:SS.sss+12:00 format (95% data loss). This failure may explain why GBIF did not delete the earlier versions of the 1186 duplicated records (see Methods), as both the earlier and later versions of these records have *modified* entries in YYYY-MM-DDTHH:MM:SS.sss+12:00 format.

Other major losses were in the *eventDate* and *dateIdentified* fields and were sometimes inconsistent. Here are details of an example: the MV-ALA dataset contains 341,693 correctly formed entries in *eventDate_raw*. These include 13,815 interval dates. The entire interval date was excluded when the format was YYYY-MM-DD/YYYY (23 records) or YYYY-MM-DD/YYYY-MM (32). The earlier date in the interval (only) was accepted from the formats YYYY-M-D/D (1 record), YYYY-MM-DD/DD (11037 plus an exception, see below), YYYY-MM-DD/MM-DD (2317) and YYYY-MM-DD/YYYY-MM-DD (404 plus an exception). One of the two exceptional exclusions was the entry “2006-09-02/2005-11-20”, which is malformed as an interval date. Its exclusion suggests that ALA tested interval dates before deleting the later date. The second exclusion was “1943-06-20/21” for *catalog Number* C.95257 (https://biocache.ala.org.au/occurrences/5966a91c-b333-4781-924f-92f1f6f57919; accessed 2018-02-21). The same interval date (“1943-06-20/21”) was accepted for four other records, e.g. C.95256 (https://biocache.ala.org.au/occurrences/b78583d0-4195-4a65-a733-6445394e7bd2; accessed 2018-02-21). Among non-interval dates, ALA excluded YYYY (98812 records) and YYYY-MM (51412), while accepting YYYY-M-D (2), YYYY-MM-D (1) and YYYY-M-DD (1). All but 258 of 177,650 valid YYYY-MM-DD dates were accepted. All 258 appear to be well-formed, and as with “1943-06-20/21” above, ALA accepted and excluded the same YYYY-MM-DD in different records. For example, “1985-10-06” was rejected in C.364665 (https://biocache.ala.org.au/occurrences/16a2f2ee-a330-4a4a-8a89-b1450f00f270; accessed 2018-02-21) but accepted in C.441797 (https://biocache.ala.org.au/occurrences/dcdc015f-363d-4d5e-a532-2a8a4988ee20; accessed 2018-02-21). In summary, ALA filtered out 150,537 of all valid *eventDate_raw* entries in the MV dataset (44 % loss) and accepted only the starting date in non-excluded interval dates.

ALA also had processing losses in fields containing names of persons and organisations. In the AM dataset, the original *recordedBy_raw* field has 236,855 entries in a range of formats. These include both “name” (e.g. “A.C. & J.E.Miller”) and “reverse name” (e.g. “Abbott, E.”) entries, as well as oddities such as “aborigines” and “Unknown (Sea Gypsies)”. ALA excluded no *recordedBy_raw* entries and processed 66,900 entries without change. Many of the 169,955 entries changed by ALA (72% of the total) were not processed successfully. Some systematic failures are listed below. The most significant errors were the replacement of a valid string by “null”, which occurred in 7,596 entries (3.2% of total), and the data losses associated with conjunction failure (see below), which I did not tally.

**null** A name string was replaced by the word “null”, e.g. “A.Musgrave & E.LeG.Troughton” processed as “Musgrave, A.|null”.

**conjunction failure** Conjunctions and separators were replaced with pipes (“|”). The results were variably successful, with some pipes separating two recorders correctly, e.g. “J.& D.Freeman” processed as “Freeman, J.|Freeman, D.” A large number (not tallied) of entries were incorrectly piped, e.g. “J.Brazier & G.Rossiter” processed as “Rossiter, J.|Rossiter, G.” and “J.Paxton & M.McGrouther” processed as “Mc, J.|Mc, M.”. Conjunction failures also saw institutional affiliations become separate recorders, as with “Kessner, Mr. Vince - Australian Museum - Malacology” processed as “Kessner, V. Vince|Australian Museum|Malacology”.

**initial reversal** The order of the first name and middle initial were reversed, e.g. “Harvey, Michael S.” processed as “Harvey, S. Michael”.

**added initial** The initial of the first name is added, e.g. “Houghton, Noel” processed as “Houghton, N. Noel”.

**initial comma** A comma was placed at the beginning of the name(s), e.g. “B.M.R.” processed as “, B.M.R.” and “W.F.& J.M.Ponder & T.Habe” as “, W.F.|Ponder, J.M.|Habe, T.”.

**surname ending** The string “and” at the end of a surname was processed as the conjunction “and”, e.g. “M. Crossland” replaced by “, M. Crossl|”.

GBIF processed most names without change in the *recordedBy* and *identifiedBy* fields, but excluded 443 *recordedBy* entries representing 119 unique name strings in the MV dataset. A check of several of the excluded entries shows that they were accepted by GBIF in other records. For example, “Peter K. Lillywhite - Museum Victoria” was excluded in four records but accepted in 233 others. This inconsistent processing resulted in a loss of <1% of valid data items.

### Other data issues

While checking for taxon name changes and data losses, I incidentally noted several other unwelcome results of data processing, described here.

In the NZAC dataset, *verbatim.txt* has the string “not identified on slide” in the *scientificName* field for *catalog Number* NZAC02015964, and the other taxonomic fields are empty. GBIF matched this string with the fictitious genus *Not* Chan, 2016, to which occurrence records from other datasets had also been matched, for entries such as “Not naturalised in SA sp.” and “Not listed”. I published this processing error in a post to the *iPhylo* blog on 24 January 2018 (http://iphylo.blogspot.com.au/2018/01/guest-post-not-problem.html). The “*Not* Chan, 2016” page was subsequently removed from the GBIF website and the NZAC “not identified on slide” record is now processed as “incertae sedis” (https://www.gbif.org/occurrence/1315606681; accessed 2018-03-01).

In the MV dataset, ALA processed *geodeticDatum_raw* entries to *geodeticDatum* inconsistently and with errors. Of the “WGS84” entries, 341539 were correctly processed to the equivalent EPSG:4326, while 652 were deleted. Also processed to EPSG:4326 were 203 “AGD66” entries, for which the correct equivalent is EPSG:6202; the difference on the ground is ca 200 m.

ALA processes locality text fields on the basis of the supplied decimal latitude and longitude values, disregarding original values in text fields. If the supplied coordinates are incorrect, ALA’s processing sometimes adds incorrect text data. In the AM dataset, *catalog Number* C.500744 is from Roma Gorge in Australia’s Northern Territory but was assigned to Mozambique (“-23.63805 32.41804” supplied instead of -23.63805 132.41804) and C.429548.002 from near Newcastle Waters in the Northern Territory was assigned to Namibia (“-17.25750 13.45444” instead of -17.25750 133.45444). In both cases *country_raw* is “Australia” and *stateProvince_raw* is “Northern Territory”. In the MV dataset, ALA assigned Coral Bay (*country_raw* = “Australia”, *stateProvince_raw* = “Western Australia”) to Botswana (“-23.14 23.14” supplied instead of -23.14 113.77; *catalog Number* HET5323), and Clifton Downs Station (“Australia” and “New South Wales”) to South Africa (“-29.61 29.61” instead of -29.61 142.57; HET4586).

## Discussion

Users can download from ALA and GBIF, as I did, sets of occurrence records containing both original and processed data items. However, both ALA and GBIF recommend smaller downloads containing only processed records. As noted above, these processed records can have blanks where data was originally provided, can feature taxon names different from the ones used by the data provider, and can contain processing errors. The user of a recommended ALA or GBIF download has no way of knowing which names have been changed or which data items lost, other than to go to the ALA and GBIF websites and look for original vs processed data differences for individual records, or investigate individual data quality “flags” and “assertions”.

I do not know the extent to which data providers are informed by aggregators about changes made in the data supplied, or whether providers, under the terms of legal arrangements with aggregators, can ask that changes be reversed. Museums and herbaria might be particularly concerned at the confusion in processed records regarding the identity of type specimens. These questions were addressed in general terms in a published response to an earlier audit of mine which looked at the quality of occurrence data as provided to aggregators (Mesibov 2013):

“Some data providers encourage the ALA to make corrections to the provider’s records (for provider and ALA). Other data providers would withdraw their support if similar changes were attempted on their data by the ALA. Feedback from the ALA to a data provider may result in immediate corrections (and data propagation) while in other cases, the provider has no resources to resolve an issue. There is no single process here that will work effectively in all circumstances.” (Belbin et al. 2013).

Note, however, that these comments referred to problems in data as provided, not to problems generated at aggregator level, as reported here.

It is a little surprising that GBIF asks users to cite their downloaded data as authored by the provider (See Methods, Data sources), and that ALA likewise asks (in each download’s *citation.csv* file) that data be cited as records from the provider. Clearly this is not the case for processed data. It would be more correct to say that aggregated data are made available as the combined work of provider and aggregator, and that the aggregator is solely responsible for any differences between original and processed data.

Some of the processing problems noted in this paper are the result of programming errors. Given the results reported here, it seems unlikely that ALA and GBIF programming staff or contractors have systematically compared original and processed data to look for problems in selected fields, as I did. I am also aware that issues raised on the ALA GitHub site (https://github.com/AtlasOfLivingAustralia) by staff and users can remain open for long periods (e.g. https://github.com/AtlasOfLivingAustralia/ala-downloads/issues/17; accessed 2018-03-03), and that closed issues may still be open, e.g. the lack of a processed *locality* field, which was said to have been corrected two months before I downloaded ALA data for audit (https://github.com/AtlasOfLivingAustralia/ala-downloads/issues/14; accessed 2018-03-03). The apparent failure of ALA and GBIF to monitor processing output is both surprising and disappointing. It is particularly surprising that data loss in processing of dates remains a significant problem, five years after a careful analysis of GBIF date losses was published by Otegui et al. (2013a).

Data loss may be the result of programming “bugs”, but both ALA and GBIF regard the replacement of originally supplied taxon names as a feature of their aggregation protocols. This view has its critics (Franz and Sterner 2018), and it is hard to understand how losing a taxon name through fail-matching or up-matching improves an occurrence record. Otegui et al. (2013b) attribute some failed matching to inadequate provision of higher-taxon information in original records. The remarkable lack of agreement between ALA and GBIF on replacement names for the same original names highlights another problem: reference classifications differ. The Catalogue of Life Plus project (https://github.com/Sp2000/colplus; accessed 2018-03-01) will attempt to replace differing reference classifications with a single “consensus” one for aggregator use, but it seems unlikely that the result will be universally adopted by data providers, let alone by the taxonomists who supply scientific names to museums and herbaria.

Three recommendations could be made to improve the taxonomic usefulness of aggregated occurrence records. A simple, easily implemented one is for aggregators to include both original and processed taxonomic data items in each record, from *scientificName* and all higher taxon fields. A second improvement would be for aggregators to employ, as Franz and Sterner (2018) propose, multiple reference classifications, so that each original taxon name could be seen in a range of taxonomic contexts. A third possible advance would be for aggregators to construct sets of related name strings, so that each original name can be seen by the user as one element in a group of variants, synonyms and fuzzy matches – a “see also” function in searches and record selection for download.

There have been a number of recent studies which question the quality of aggregated occurrence records (see references in Franz and Sterner 2018), but Sikes et al. (2016) defend GBIF and other aggregators by saying that all data need vetting and processing and that the quality of aggregated data is the collective responsibility of the biodiversity data community. These are truisms. It is also obvious that aggregators should not lose or confuse the data they are provided with, and the present audit suggests that both ALA and GBIF could do better. End-users of aggregated occurrence records would be wise to download both the original and the processed datasets, and to check carefully for data losses and taxon name replacements.
